# Axitinib induces DNA damage response leading to senescence, mitotic catastrophe, and increased NK cell recognition in human renal carcinoma cells

**DOI:** 10.18632/oncotarget.5768

**Published:** 2015-10-14

**Authors:** Maria Beatrice Morelli, Consuelo Amantini, Matteo Santoni, Alessandra Soriani, Massimo Nabissi, Claudio Cardinali, Angela Santoni, Giorgio Santoni

**Affiliations:** ^1^ School of Pharmacy, Experimental Medicine Section, University of Camerino, Camerino, Italy; ^2^ Department of Molecular Medicine, Sapienza University, Rome, Italy; ^3^ School of Biosciences and Veterinary Medicine, University of Camerino, Camerino, Italy; ^4^ Department of Medical Oncology, AOU Ospedali Riuniti, Polytechnic University of the Marche Region, Ancona, Italy

**Keywords:** renal cell carcinoma, tyrosine kinase inhibitors, cell senescence, mitotic catastrophe, NKG2D ligands

## Abstract

Tyrosine kinase inhibitors (TKIs) including axitinib have been introduced in the treatment of renal cell carcinoma (RCC) because of their anti-angiogenic properties. However, no evidence are presently available on a direct cytotoxic anti-tumor activity of axitinib in RCC.

Herein we reported by western blot analysis that axitinib treatment induces a DNA damage response (DDR) initially characterized by γ-H2AX phosphorylation and Chk1 kinase activation and at later time points by p21 overexpression in A-498 and Caki-2 RCC cells although with a different potency. Analysis by immunocytochemistry for the presence of 8-oxo-7,8-dihydro-2′-deoxyguanosine in cellular DNA and flow cytometry using the redox-sensitive fluorescent dye DCFDA, demonstrated that DDR response is accompanied by the presence of oxidative DNA damage and reactive oxygen species (ROS) generation. This response leads to G2/M cell cycle arrest and induces a senescent-like phenotype accompanied by enlargement of cells and increased senescence-associated β-galactosidase activity, which are abrogated by N-acetyl cysteine (NAC) pre-treatment. In addition, axitinib-treated cells undergo to cell death through mitotic catastrophe characterized by micronucleation and abnormal microtubule assembly as assessed by fluorescence microscopy.

On the other hand, axitinib, through the DDR induction, is also able to increase the surface NKG2D ligand expression. Accordingly, drug treatment promotes NK cell recognition and degranulation in A-498 RCC cells in a ROS-dependent manner.

Collectively, our results indicate that both cytotoxic and immunomodulatory effects on RCC cells can contribute to axitinib anti-tumor activity.

## INTRODUCTION

Renal cell carcinoma (RCC) accounts for 2-3% of all malignancies, with approximately 84,400 new cases and 34,700 cancer-related deaths in Europe in 2013 [1]. Almost one third of the patients present metastatic disease at diagnosis and another 20% develop metastases after nephrectomy [2, 3].

Angiogenesis is critical for sustaining RCC growth and haematogenous dissemination [4]. Tyrosine kinase inhibitors (TKIs) targeting vascular endothelial growth factor receptor (VEGFR), such as sunitinib, sorafenib, pazopanib and axitinib, the anti-VEGF antibody bevacizumab and the mammalian target of rapamycin (mTOR) inhibitors everolimus and temsirolimus, have been sequentially approved by the US Food and Drug Administration (FDA) [5-12].

Axitinib is a potent and selective inhibitor of VEGFR 1, 2, and 3 approved by FDA in 2012 for the treatment of patients with metastatic RCC (mRCC) after failure of one prior systemic therapy. The European Medicines Agency has approved the use of axitinib in 2015 for the treatment of advanced renal carcinoma after failure of prior treatment with sunitinib or interleukin 2 (IL-2) [13]. Its use as first-line therapy for advanced or mRCC was also reported [14, 15].

In experimental models, axitinib produces a dose-dependent blockade of VEGFR-2 phosphorylation, reduction of vascular permeability and angiogenesis, and induction of apoptosis, providing evidence for therapeutic potential [16]. Moreover, in murine RCC xenografts, axitinib augments CD8^+^ T cell-mediated antitumor activity against renal carcinoma via a STAT3-dependent reversal of myeloid suppressor cells (MDSC) accumulation in the spleens and tumor beds [17].

Agents that cause genotoxic stress or DNA-replication inhibitors have been recently shown to activate the DNA damage response (DDR) as well as to increase the expression of stress-induced NKG2D and DNAX accessory molecule-1 (DNAM-1) ligands recognized by the innate immune system [18]. DDR to genotoxic insults involves a class of protein kinases, including ATM, ATR, and DNA-dependent protein kinases, followed by activation of Chk1 and Chk2 kinases that causes temporal cell cycle arrest, and promotes assembly of DNA repair complexes at the damaged sites on chromosomes [19-21]. *In vivo* activation of Chk1 requires phosphorylation on both Ser-345 and Ser-317 [22]. Cell cycle arrest can then lead to different cellular programs including senescence, apoptosis and mitotic catastrophe [23, 24].

Beyond its effects on angiogenesis, axitinib has been recently shown to modulate the function of immune effector cells that play an important role in the control of RCC development, progression and drug response [25, 26]. RCC exhibits a prominent immune cell infiltrate consisting of T cells, dendritic cells (DCs), macrophages and natural killer (NK) cells.

NK cells represent one of the main effectors of the immunosurveillance against tumors [27, 28]. NK cell activity depends on the interplay between inhibitory receptors for major histocompatibility complex (MHC) class I molecules and activating receptors, such as NKG2D and DNAM-1 that operate in concert to induce the elimination of tumor cells [29, 30]. Human NKG2D belongs to C-type lectin-like receptor family and recognizes MHC I-related molecules MICA/B and ULBPs (UL16-binding proteins) [31-33]. NKG2D is expressed not only on NK cells, but also on γδ T cells, CD8^+^ T cells, and a subset of CD4^+^ T cells. The expression of NKG2D ligands is largely confined to virus-infected, tumor, and stressed cells [31]. DNAM-1 is a transmembrane glycoprotein constitutively expressed on the majority of T cells, NK cells, and macrophages. DNAM-1 ligands, namely nectin-2 (Nec-2, CD112) and the poliovirus receptor (PVR, CD155), have been initially described as adhesion molecules and only recently they have been found on a variety of tumors and virus-infected cells [33-35].

In this study, we demonstrated the ability of axitinib treatment to trigger DNA damage response, cell cycle arrest and senescence, and mitotic catastrophe in RCC cells. In addition, we further evaluated axitinib ability to increase NKG2D and DNAM-1 ligand surface expression and to enhance NK cell recognition and activity against RCC cells.

## RESULTS

### Axitinib inhibits RCC cell viability in a dose and time-dependent manner

**Figure 1:**
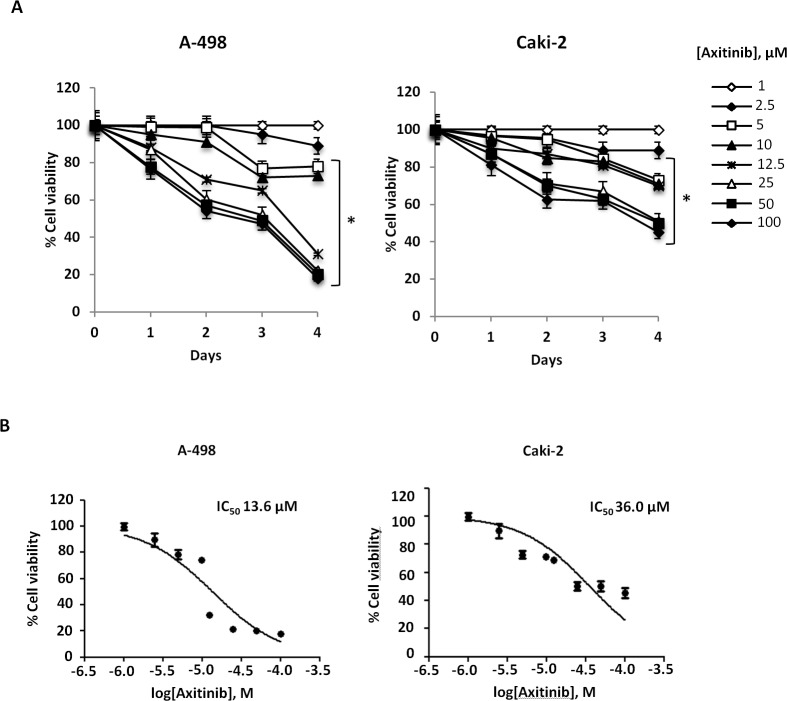
Axitinib inhibits RCC cell viability in a dose and time-dependent manner **A.** A-498 and Caki-2 RCC cell lines were cultured up to 96 h with different doses of axitinib. Cell viability was determined by MTT assay. Data shown are expressed as mean ± SD of three separate experiments; *p < 0.01 *vs* vehicle-treated cells. **B.** RCC cell lines were cultured for 96 h with different doses of axitinib. Cell viability was determined by MTT assay. Data shown are expressed as mean ± SE of three separate experiments.

We first evaluated the effects of axitinib on cell viability in A-498 and Caki-2 RCC lines by performing dose-response and time-course analyses (Figure 1). Axitinib inhibited the growth of RCC lines, with IC50 values of 13.6 μM for A-498 and 36 μM for Caki-2 cells after 96 h of treatment, indicating that Caki-2 cells are more resistant to axitinib-mediated cytotoxic effects. The lowest effective dose of axitinib inducing growth inhibition (12.5 μM for A-498 and 25 μM for Caki-2 cells after 96 h treatment) was used for the subsequent experiments.

### Axitinib triggers DDR associated with oxidative DNA damage in RCC cells

**Figure 2:**
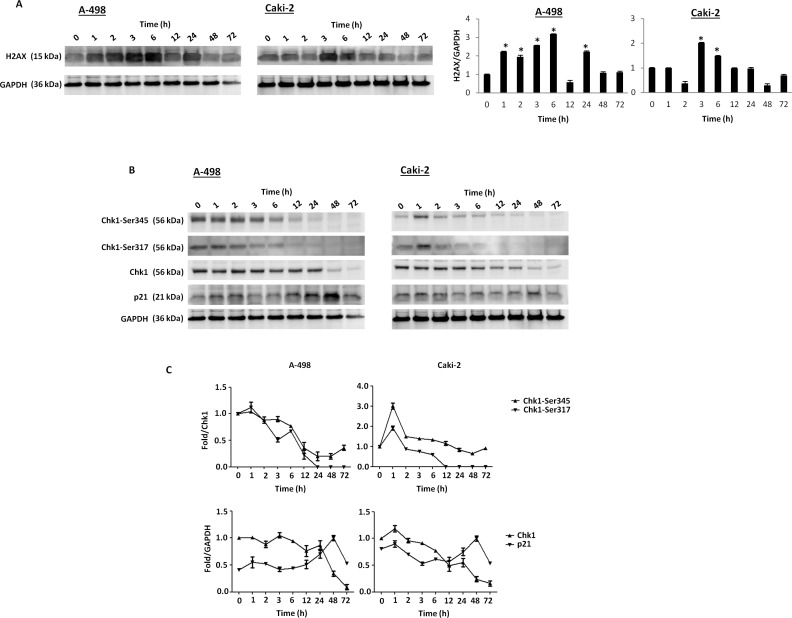
Axitinib triggers DDR in RCC cells **A.** Western blot analysis and densitometry quantification of H2AX protein levels in RCC cells cultured for up to 72 h in the presence of axitinib (12.5 μM in A-498 and 25 μM in Caki-2). H2AX densitometry values were normalized to GAPDH used as loading control. Blots are representative of one of three separate experiments, *p < 0.01 treated *vs* untreated cells. **B.** Western blot analysis of Chk1-Ser345, Chk1-Ser317, Chk1 and p21 protein levels in RCC cells cultured for up to 72 h as above described. Blots are representative of one of three separate experiments. **C.** Quantitative representation of the experiment reported in panel B. Chk1 and p21 densitometry values were normalized to GAPDH used as loading control. The Chk1-Ser345 and Chk1-Ser317 protein levels were determined with respect to Chk1 levels. For Chk1-Ser345, Chk1-Ser317 and Chk1, the initial protein levels were taken as 1. For p21, the maximal p21 protein levels were also taken as 1.

To evaluate whether axitinib treatment could trigger DDR in RCC cells, we initially investigated the presence of γ-H2AX (H2AX), a phosphorylated variant of histone 2A that is associated with DNA double-strand breaks [36]. Interestingly, western blot analysis revealed strong induction of the DNA damage marker in both RCC cell lines, being more rapid and sustained in A-498 cells (Figure 2A). γ-H2AX induction was accompanied by Ser317- and Ser345-Chk1 phosphorylation already after 1 h exposure to axitinib and persisting at later points only in A-498 cells (Figure 2B, 2C). Later at 12 h after treatment, a progressive overexpression of p21 that paralleled the decline of Ser345- and Ser317-Chk1 activation and Chk1 protein levels, was mainly observed in A-498 cells (Figure 2B, 2C).

**Figure 3:**
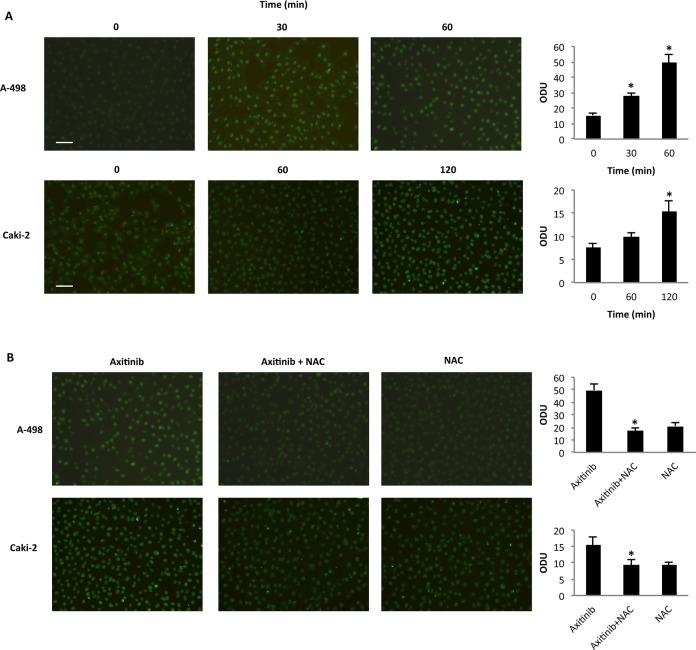
Axitinib triggers oxidative DNA damage in RCC cells **A.** Immunofluorescence analysis of 8-oxo-dG in A-498 and Caki-2 RCC cells treated with axitinib (12.5 μM and 25 μM, respectively) for different times. Optical Density Units (ODU) were calculated on ten random fields. Data shown are representative of one of three separate experiments, *p < 0.01 *vs*. untreated cells. Bar: 100 μM. **B.** Immunofluorescence analysis of 8-oxo-dG in A-498 cells treated with axitinib 12.5 μM for 60 min and in Caki-2 cells treated with axitinib 25 μM for 120 min alone or pretreated with NAC (10 mM for 1 h). ODU were calculated on ten random fields. Data shown are representative of one of three separate experiments, *p < 0.01 *vs*. axitinib treated cells. Bar: 100 μM.

In addition, immunofluorescence analysis with 8-oxo-7,8-dihydro-2-deoxyguanosine (8-oxo-dG), a marker of oxidative DNA damage [37], in cells treated for different times with axitinib alone or in combination with the antioxidant NAC, indicated that axitinib induces oxidative DNA damage in a time-dependent manner, being more rapid and prominent in A-498 cells (Figure 3A); this response was reverted by pre-treatment of RCC cells with the antioxidant NAC (Figure 3B).

### Axitinib induces G2/M arrest and cell senescence in RCC cells

**Figure 4:**
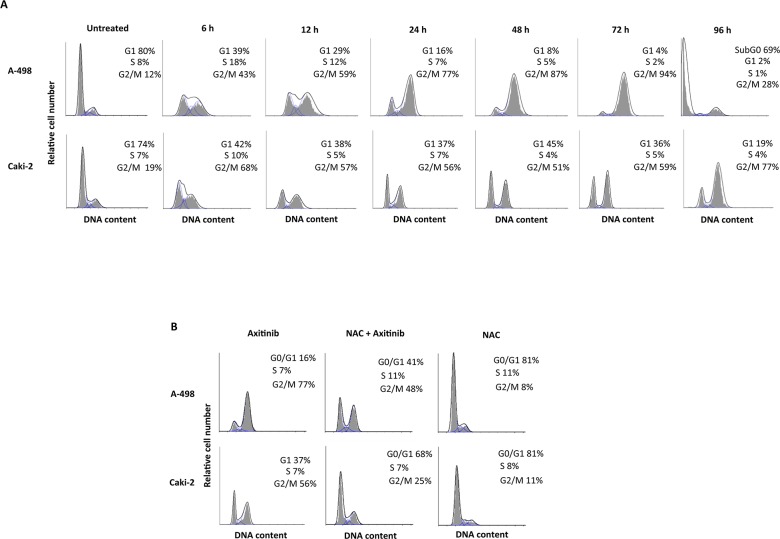
Axitinib induces cell cycle arrest in ROS-dependent manner **A.** Cell cycle analysis in A-498 cells treated with axitinib 12.5 μM and in Caki-2 cells treated with axitinib 25 μM for the indicated times. **B.** Representative cell cycle distribution in A-498 and Caki-2 cells pretreated or not with NAC (10 mM for 1 h) before axitinib treatment for 24 h.

We then evaluated whether axitinib treatment could result in changes in cell cycle. Thus, we performed cell cycle experiments in the presence of axitinib for 96 h. We observed that treatment of RCC cells induced a significant decrease of G0/G1 phase cell population already at 6 h and this decrease was accompanied by a parallel and progressive increase of G2/M-phase cell population until 72 h (Figure 4A). Again, the axitinib effects were more potent in A-498 cells as compared to Caki-2 cells. In addition, pre-treatment of RCC cells with NAC reverted the axitinib-induced effects on cell cycle at any experimental time point tested (Figure 4B, and data not shown).

**Figure 5:**
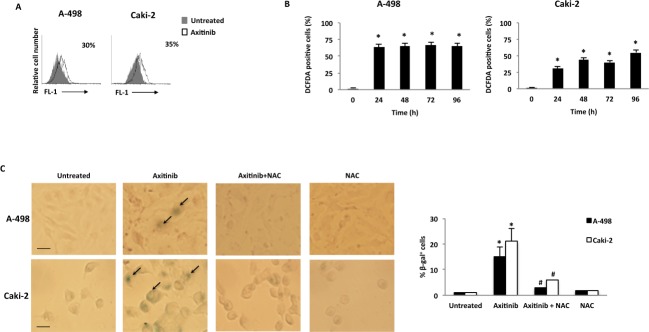
Axitinib induces cellular senescence in RCC cells in a ROS-dependent manner **A.** Representative flow cytometric profiles of RCC cells untreated or treated with axitinib for 72 h, then stained with C_12_FDG, a fluorogenic substrate for SA-β-galactosidase before analysis. Data represent the percentage of positive cells. **B.** ROS generation in RCC cell lines treated with axitinib for the indicated times. Cells were stained with DCFDA before flow cytometric analysis. Data are expressed as percentage of DCFDA positive cells with respect to untreated cells; *p < 0.01 treated *vs.* untreated cells. **C.** Cellular senescence was assessed in A-498 cells treated with axitinib 12.5 μM and in Caki-2 cells treated with axitinib 25 μM for 72 h, with or without pretreatment with NAC 10 mM for 1 h, by detection of SA-β-galactosidase activity using cytochemistry. Arrows indicate blue-stained positive cells. Graph represents the percentage of β-galactosidase^+^ cells calculated on ten random fields. Data shown are representative of one of three separate experiments, *p < 0.01 *vs*. untreated cells. ^#^*p* < 0.01 *vs*. axitinib-treated cells. Bar: 25 μM.

An accumulation of RCC cells with enlarged and flattened morphology was observed at 48 h after axitinib treatment by microscopy and biparametric (forward scatter, FSS *vs* side scatter, SSC) cytofluorimetric analysis (Supplementary Figure 1). Since these morphological changes are reminiscent of a senescent phenotype, we analyzed the presence of senescence-associated β-galactosidase (SA-β-gal) activity, in axitinib-treated cells [38, 39]. Seventy-two hours after axitinib treatment, increased levels of SA-β-gal activity were detected in RCC cells stained with the fluorescent β-galactosidase substrate, C_12_FDG as determined by flow cytometry. (Figure 5A).

Recent studies have suggested that reactive oxygen species (ROS) generation by anticancer drug treatment can stimulate cellular senescence [40]. Thus, we evaluated the generation of ROS in RCC cells by flow cytometry using the general redox-sensitive fluorescent dye, DCFDA. As shown in Figure 5B, axitinib stimulated intracellular generation of ROS that was evident at 24 h after exposure, being more rapid and sustained in A-498 cells. These results were also supported by cytochemical assessment of SA-β-gal activity that revealed a significant reduction in the percentage of blu-stained axitinib-treated senescent cells after pretreatment with NAC (Figure 5C).

### Axitinib induces mitotic catastrophe in RCC cells

**Figure 6:**
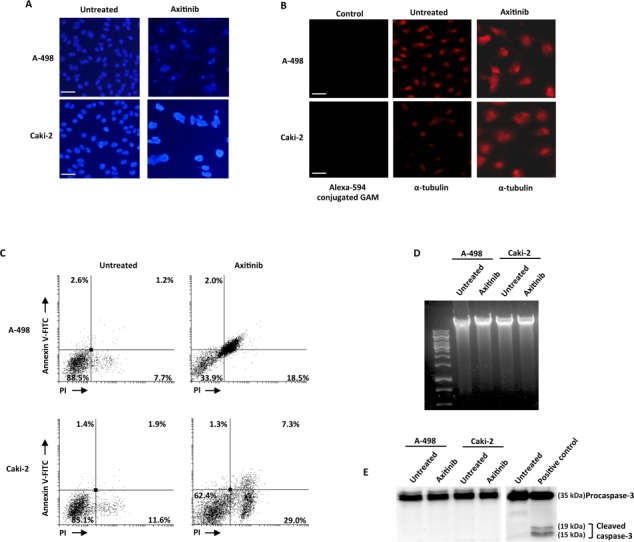
Axitinib induces mitotic catastrophe in RCC cells **A.** Nuclei of RCC cells untreated or treated with axitinib for 96 h were stained with Hoechst 33258 and then analyzed on ten random fields. Cells were observed under a fluorescence microscope. Bar: 50 μM. **B.** Representative images of RCC cells treated as above described, and then immunostained with anti-α-tubulin antibody. Bar: 50 μM. **C.** RCC cells were cultured for 96 h with axitinib. Flow cytometric analysis was performed on treated cells by Annexin V-FITC and PI double-staining. Data represent the percentage of PI and/or Annexin V positive cells. **D.** Representative agarose gel electrophoresis of DNA extracts obtained from untreated or axitinib treated cells at 96 h for assessment of DNA fragmentation. **E.** Representative immunoblot of caspase-3 in RCC cells treated as above described.

Mitotic catastrophe is a non-apoptotic cell death resulting from cell cycle arrest and abnormal mitosis, usually ending in the formation of large cells with multiple micronuclei [41]. Thus we decided to study whether axitinib treatment could also result in mitotic catastrophe in RCC cells, by assessing the changes in nuclear morphology using Hoechst 33258 staining. Increased number of micronuclei was observed at 96 h in both RCC cell lines (Figure 6A). In addition, by examining the expression of α-tubulin, abnormal microtubule assembly was found in axitinib-treated RCC cells at 96 h after treatment (Figure 6B). To further support mitotic catastrophe as the mode of death, FITC-conjugated Annexin V/PI and cytofluorimetric analysis, agarose gel electrophoresis for DNA fragmentation and western blot analysis for caspase-3 activity were performed in untreated or axitinib-treated cells. Axitinib treatment resulted in an increased percentage of cells undergoing necrotic-like death (Annexin V^−^/PI^+^) and secondary necrosis (Annexin V^+^/PI^+^) upon drug exposure in both RCC cell lines, being the frequency of dead cells higher and the cell death program more advanced in A-498 cells as compared to Caki-2 cells (Figure 6C); moreover, neither DNA fragmentation (Figure 6D) or caspase-3 activation (Figure 6E) was evidenced in axitinib-treated RCC cells.

Taken together, these results indicate that axitinib induces cell death in RCC cells through mitotic catastrophe.

### Axitinib preferentially increases NKG2D ligand expression in senescent RCC cells

**Figure 7:**
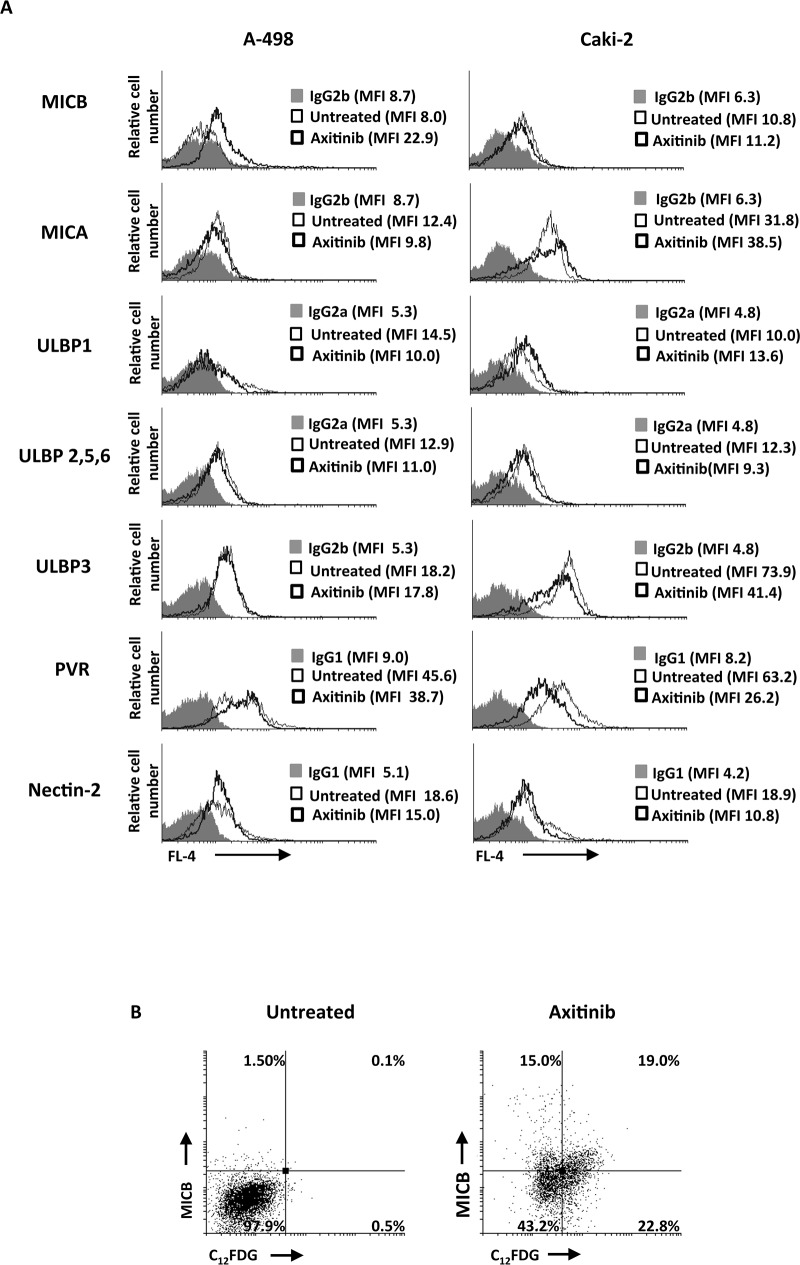
Modulation of NKG2D and DNAM-1 ligand expression on the RCC cell lines by axitinib treatment **A.** MICB, MICA, ULBP1, ULBP2, 5, 6 and ULBP3, PVR and nectin-2 surface expression was analyzed by flow cytometry on RCC cells treated for 72 h with axitinib or untreated. Light lines represent ligand expression in untreated cells, dark lines represent ligand expression in axitinib-treated RCC cells, whereas gray histogram isotype controls. MFI, mean fluorescence intensity. **B.** Representative dot plots illustrating the double fluorescence MICB-APC/C_12_FDG in A-498 RCC cells treated as above described. Numbers represent the percentage of cells in each quadrant. Results are representative of 1 of 3 independent experiments.

Since chemotherapeutic agents through the activation of the DDR have been shown to enhance the expression of NKG2D and DNAM-1 ligands on human multiple myeloma cells in a ROS-dependent manner [18], we investigated whether axitinib treatment could modulate the expression of the ligands for NKG2D and DNAM-1 activating NK receptors on A-498 and Caki-2 RCC lines, and the involvement of ROS signaling in this event. To this aim we firstly evaluated the basal expression of NKG2D (MICA, MICB, ULBP1, 2, 3, 5, 6) and DNAM-1 (PVR and nectin-2) ligands on A-498 and Caki-2 RCC cells by immunofluorescence and cytofluorimetric analysis. Both cell lines constitutively express MICA and ULBP family NKG2D ligands, and PVR and nectin-2 DNAM-1 ligands, but with a different profile (Figure 7A). Seventy-two hour treatment of RCC cells with axitinib induced MICB expression in A-498 cells, and increased the expression of ULBP1 and MICA in Caki-2 cells, respectively (Figure 7A). In addition, down-regulation of nectin-2 and PVR DNAM-1 ligands was observed in Caki-2 cells upon drug exposure (Figure 7A). Moreover, we found that MICB was preferentially expressed on FDG-positive A-498 cells undergoing senescence as demonstrated by double immunofluorescence and flow cytometry (Figure 7B).

**Figure 8:**
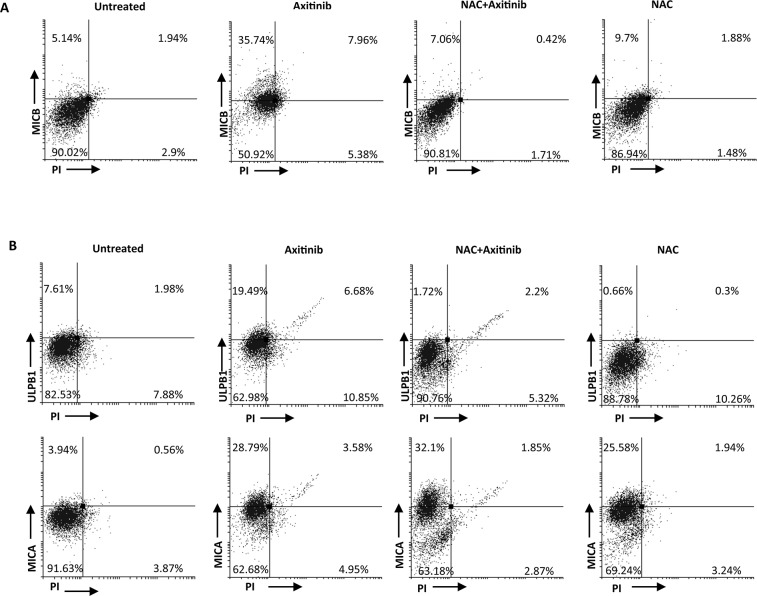
ROS-dependent axitinib-induced increase of NKG2D ligand expression in the RCC cell lines **A.** Increase of MICB surface expression was analyzed by flow cytometry on A-498 RCC cell line treated with axitinib for 72 h with or without pretreatment with NAC 10 mM for 1 h. Representative dot plots illustrate the double fluorescence ligands-APC/PI. Numbers represent the percentage of cells in each quadrant. Data are representative of 1 of 4 independent experiments. **B.** Increase of MICA and ULBP1 surface expression was analyzed by flow cytometry on Caki-2 RCC cells treated with axitinib for 72 h with or without pretreatment with NAC 10 mM for 1 h. Representative dot plots illustrate the double fluorescence ligands-APC/PI. Numbers represent the percentage of cells in each quadrant. Data are representative of 1 of 4 independent experiments.

We next investigated the expression of MICB, MICA and ULBP1 on PI^−^ A-498 and Caki-2 RCC cells, respectively, in the presence of anti-oxidant NAC. Exposure of RCC cells to NAC resulted in complete inhibition of MICB and ULBP1 expression in A-498 and Caki-2 cells, respectively (Figure 8A and 8B), whereas NAC pretreatment did not significantly reduce axitinib-induced increase of MICA expression on Caki-2 cells that exhibited a weaker oxidative stress response.

### Enhanced NK cell degranulation upon interaction with axitinib-treated A-498 RCC cells

**Figure 9:**
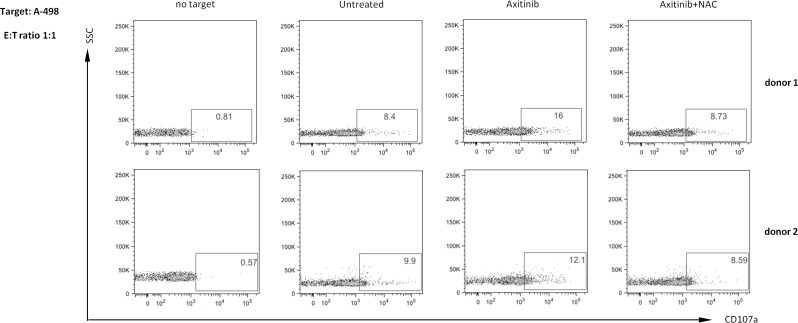
Enhanced NK cell degranulation upon interaction with axitinib-treated A-498 RCC cells is ROS-dependent A-498 RCC cells were treated with axitinib 12.5 μM for 72 h with or without pretreatment with NAC 10 mM for 1 h, and then incubated with freshly isolated human peripheral blood NK cells from two different healthy donors at 1:1 ratio for 2 h. Results are expressed as percentage of CD107a^+^ NK cells.

Based on these findings, we evaluated whether axitinib would increase NK cell degranulation upon interaction with drug-treated RCC cells. The expression of the lysosomal marker CD107a, which correlates with NK cell cytotoxicity [42] was evaluated by immunofluorescence and flow cytometry analysis by gating on CD56^+^ human peripheral blood NK cells contacting treated or untreated RCC cells used as target. The expression of CD107a on NK cells from two different healthy donors contacting axitinib treated-RCC target cells, revealed that drug-treated A-498 RCC cells more efficiently triggered NK cell degranulation as compared to untreated cells, and this enhancement is completely blocked by NAC pretreatment, in parallel with inhibition of MICB induction (Figure 9). Conversely, no significative changes in CD107a expression were observed on NK cells contacting axitinib-treated Caki-2 cells (Supplementary Figure 2), likely attributable to the lower responsiveness of these cells to the axitinib-exerted activity.

## DISCUSSION

TKIs and in particular axitinib have been recently approved for the treatment of advanced mRCC, mainly for their anti-angiogenic properties. Herein, we provide evidence indicating that axitinib anti-tumor activity can be also the result of its ability to induce DDR, cellular senescence and mitotic catastrophe, and to enhance the recognition of RCC cells by innate immune effector cells through the modulation of activating ligands.

In particular, we found that treatment of RCC with suboptimal doses of axitinib triggers a DDR evidenced by increased levels of H2AX, Ser317- and Ser345-Chk1 phosphorylation and DNA oxidation, leading to cell cycle arrest at G2/M phase and cellular senescence. At later time points, overexpression of the cell cycle inhibitor p21 was observed and paralleled decreased levels of Chk1 activation and depletion. In this regard, reactivation of a senescence program in PC-3 prostate cancer cells upon a retrovirus-mediated transduction of p21, was described to suppress Chk1 activation [43].

We also investigated the involvement of redox signaling on the ability of axitinib treatment to induce cellular senescence in A-498 and Caki-2 RCC cells. We found that drug exposure increases ROS generation and induces the DNA oxidation, whereas pretreatment with the antioxidant agent NAC results in impaired cell cycle arrest and decreased percentage of SA-β-gal positive cells.

Similarly to our study, treatment of gastric cancer cells with axitinib was reported to induce a senescent phenotype characterized by increased cell size, expression of SA-β-galactosidase and arrest in G2 cell cycle phase [44].

Cell cycle arrest and senescence are often associated with mitotic catastrophe. Chk1, the kinase that regulates the G2/M checkpoint, is also particularly important for preventing mitotic catastrophe in cells treated with DNA-damaging agents [45-47]. Mitotic catastrophe has been described as the main form of cell death induced by anticancer drugs [48, 49]. Similarly to necrosis, mitotic catastrophe shows early loss of plasma membrane integrity, with large cells containing uncondensed chromosomes [50]. Treatment with axitinib caused an increased frequency of cells undergoing necrotic-like death and secondary necrosis as well as mitotic catastrophe characterized by multinucleation, abnormal microtubule assembly, early membrane permeabilization and absence of apoptotic features such as caspase 3 activation and DNA fragmentation.

Our study provides also evidence that axitinib treatment increases the surface expression of stress-induced ligands recognised by innate immune effector cells.

Up-regulation of stress-inducible NK-cell activating ligands is preferentially associated with the onset of a senescent phenotype and arrest in the G2 phase of the cell cycle [51]. Our findings demonstrate that A-498 and Caki-2 RCC cells constitutively express, although at different levels, several NKG2D and DNAM-1 ligands. Axitinib treatment induced MICB expression in A-498 RCC cells and increased ULBP1 and MICA expression in Caki-2 cells. In addition in these latter cells, significative down-regulation of the DNAM-1 ligand PVR was observed.

We also demonstrated that induction of MICB on A-498 RCC cells and ULBP-1 on Caki-2 cells requires ROS signaling as ligand up-regulation was susceptible to NAC pretreatment. By contrast, in Caki-2 cells pretreated with NAC, no reversal of axitinib-induced increase of MICA expression was evident. Moreover, in accordance with previous evidence indicating that NKG2D and DNAM-1 ligand are expressed on tumor cells with senescent phenotype upon treatment with genotoxic agents [52], we found a preferential expression of MICB on axitinib-treated senescent A-498 RCC cells. Like axitinib, other TKIs such as sorafenib and sunitinib have been described to induce the expression of MICA/B in nasopharyngeal carcinoma cells [53], but their association with a senescent phenotype was not reported.

As far as the mechanisms underlying axitinib-induced MICB expression, a role for Signal transducer and activator of transcription 3 (STAT3) inhibition can be envisaged based on the recent evidences demonstrating that axitinib can stimulate anti-tumor immunity by down-regulating the STAT3 expression in Renca RCC cells [54], and MICA/B expression on cancer cells induced by genotoxic stress is enhanced by inhibition of STAT3 activity [55].

Our results also show increased degranulation activity in NK cells contacting axitinib-treated A-498 but not Caki-2 RCC cells. The failure of axitinib-treated Caki-2 cells to promote NK cell degranulation as compared to A-498 RCC cells may depend on the less potent and sustained DDR induced by axitinib in this tumor cell line. In addition, this result may be related to the drug-induced down-regulation of DNAM-1 ligand expression observed in these cells leading to lack of cooperative signals required for the triggering of NK cell cytotoxic program [56].

Collectively, our findings are consistent with previous evidence describing RCC susceptibility to NK cell-mediated cytotoxicity, and the presence of a high frequency of NK cells in the lymphocytic infiltrate of RCC predicting a better prognosis [57]. Moreover, interleukin-2 (IL-2) in combination with infusion of IL-2-activated NK and LAK cells has been widely employed as immunotherapeutic approach for RCC patients [58].

Based on these findings, the use of axitinib in sequential or combined strategies with other immunotherapic approaches, such as anti-programmed death-1 (PD-1) or PD-ligand 1 (PD-L1) agents, should be tested in prospective clinical trials. At this regard, a phase I study is in course to assess the safety of axitinib in combination with avelumab (MSB0010718C), an anti-PD-L1 antibody, in patients with advanced RCC (NCT02493751).

Taken together, our study first demonstrate that axitinib not only inhibits angiogenesis, but it can exert direct genotoxic effects on RCC cells by inducing cell cycle arrest and mitotic catastrophe, and activating a cellular senescence program. This direct cytotoxic effect of axitinib in RCC cells may partially explain the relevant gastrointestinal and hematologic toxicity of this agent [12].

In addition, axitinib can also display an immune-mediated antitumor activity by promoting NK cell-mediated recognition and elimination of RCC through the regulation NK activating ligand expression. A better dissection of the functions of immune cells in RCC microenvironment and of the immune-modulatory effects of TKIs will be crucial to optimize immunotherapeutic approaches in RCC advanced patients.

## MATERIALS AND METHODS

### Cell line culture and treatment

Human kidney cancer (Caki-2 and A-498) cell lines were purchased from Cell bank Interlab Cell Line Collection (ICLC, Italy) and cultured at 37°C in a humidified atmosphere of 5% CO_2_. Caki-2 cells were cultured in McCoy's 5a medium (Lonza Bioresearch, Basel, Switzerland) supplemented with 10% (v/v) heat-inactivated fetal bovine serum (FBS), 2mM L-glutamine and 100 IU/ml of penicillin, 100 μg of streptomycin (Lonza). A-498 cells were cultured in EMEM medium (Lonza) supplemented with 10% (v/v) heat-inactivated FBS 2mM L-glutamine and 100 IU/ml of penicillin, 100 μg of streptomycin and 1 mM sodium pyruvate (Lonza).

A-498 and Caki-2 cells were treated with different doses of axitinib (1, 2.5, 5.0, 10.0, 12.5, 25.0, 50.0 and 100 μM) for different times. In some experiments Caki-2 and A-498 cells were pretreated for 1 h with 10 mM of NAC, before axitinib treatment.

### Reagents

Axitinib ((Inlyta®) was kindly provided by Pfizer (New York, NY, USA). The following mouse monoclonal allophycocyanin (APC)-conjugated antibodies (Abs) were used: anti-MICB, anti-MICA, anti-PVR, anti-ULBP1, anti-ULBP 2,5,6, anti-ULBP3 and anti-nectin-2 (R&D Systems, Abingdon, United Kingdom). APC-conjugated goat affinity purified F(ab’)2 fragment to mouse IgG1, IgG2a, IgG2b were purchased from Jackson ImmunoResearch Laboratories (West Grove, PA). Mouse anti-α-tubulin and anti-p21 antibodies were purchased from Santa Cruz Biotechnology (Santa Cruz, CA). Rabbit anti-H2AX, anti-Chk1-Ser345, anti-Chk1-Ser317, anti-Chk1 and anti-caspase-3 were purchased from Cell Signaling Technology (Danvers, MA). The following secondary antibodies were used: horseradish peroxidase (HRP)-conjugated sheep anti-mouse IgG and HRP-conjugated donkey anti-rabbit IgG (GE Healthcare, Munich, Germany). Annexin V-FITC was purchased from eBioscience (Hatfield, UK). 8-oxo-7,8-dihydro-2′-deoxyguanosine (8-oxo-dG) MAb was purchased from Trevigen (Gaithersburg, MD, USA). Goat anti-mouse (GAM) Alexa Fluor 594 and 5-dodecanoylaminofluorescein di-β-D- galactopyranoside (C_12_FDG) were from Invitrogen (San Diego, CA, USA). Bafilomycin A1, dimethyl sulfoxide (DMSO, used as vehicle), Hoechst 33258, propidium iodide (PI, 1 μg/ml), ribonuclease A, 5-bromo-4-chloro-3-indolyl β-D-galactopyranoside (X-Gal), N-acetyl-L-cysteine (NAC), 3-(4,5-dimethylthiazol-2-yl)-2,5-diphenyltetrazolium bromide (MTT) and anti-glyceraldehyde-3-phosphate dehydrogenase (GAPDH)-peroxidase were from Sigma Aldrich (St. Louis, USA).

### MTT assay

The colorimetric MTT assay was used to evaluate the cell viability. Three x10^5^ RCC cells/ml were seeded into 96-well plates and cultured with different doses of axitinib for up to 96 h. At the end of treatment, 0.8 mg/ml of MTT was added to the samples and incubated for 3 h. Then the supernatants were discarded and coloured formazan crystals dissolved with 100 μl/well of DMSO, were read by an enzyme-linked immunosorbent assay reader (BioTek Instruments, Winooski, USA). Four replicates were used for each treatment.

### Cell cycle analysis

Three x10^5^ RCC cells/ml were treated with vehicle or axitinib, alone or in combination with NAC, for up to 72 h. Cells were collected and fixed in 70% ethanol and then washed with staining buffer (PBS, 2% FBS and 0.01% NaN3). Next, the cells were treated with 100 μg/ml ribonuclease A solution, incubated for 30 min at 37°C, stained for 30 min at room temperature with PI 20 μg/ml and finally analysed by flow cytometry using linear amplification.

### Western blot analysis

Cells were lysed in lysis buffer (1M Tris pH 7.4, 1 M NaCl, 10 mM EGTA, 100 mM NaF, 100 mM Na V0, 100 mM phenylmethylsulfonyl fluoride, 2% deoxycholate, 100 mM EDTA, 10% Triton X-l00, 10% glycerol, 10% SDS, 0.1 M Na4 P2 07) containing protease inhibitor cocktail (Sigma-Aldrich) by using a Mixer Mill MM300 (Qiagen, Hilden, Germany). Lysates were separated on SDS polyacrylamide gel and transferred onto Hybond-C extra membranes (GE Healthcare). Membrane were incubated overnight at 4°C in primary Abs (anti-caspase 3 1:100; anti-H2AX 1:1000, anti-Chk1-Ser345 1:1000, anti-Chk1-Ser317 1:1000, anti-Chk1 1:1000, anti-p21 1:300), followed by the incubation (room temperature, 1 h) with HRP-conjugated anti-rabbit or anti-mouse secondary Abs. Peroxidase activity was visualized with the LiteAblot ®PLUS (EuroClone, Milan, Italy) kit and densitometric analysis was carried out by a Chemidoc using the Quantity One software (Bio-Rad).

### Senescence analysis

We performed the senescence analysis by both microscope and flow cytometry. RCC cells were treated with axitinib or vehicle before performing the senescence-associated β-galactosidase (SA-β-Gal) assay. Cells were then fixed for 5 min at room temperature in 3% formaldehyde and incubated overnight at 37°C without CO_2_ with fresh SA-β-Gal stain solution: 1 mg/mL 5-bromo-4-chloro-3-indolyl β-D-galactoside (X-Gal), 150 mM NaCl, 2 mM MgCl_2_, 40 mM citric acid, 5 mM sodium phosphate (pH 6.0), 5 mM potassium ferrocyanide, and 5 mM potassium ferricyanide. Senescent cells were identified as blue-stained cells by standard light microscopy. Photographs were acquired and analyzed by an Olympus BX51 microscope (Hamburg, Germany) using magnification 40x.

Relatively to flow cytometry, we performed the assay using the fluorogenic substrate C_12_FDG. Drug-treated cells were incubated 1 h at 37°C and 5% CO_2_ with 100 nM bafilomycin A1 in culture medium to induce lysosomal alkalinization at pH 6 and, then, for 1 h with 33 μM C_12_FDG. Samples were immediately analyzed using FACScan cytofluorimeter using the CellQuest software. The C_12_-fluorescein signal was measured on the FL-1 detector, and β-galactosidase activity was estimated using the median fluorescence intensity (MFI) of the population.

### Annexin V and PI staining

Cell death was evaluated using Annexin V-FITC and PI staining followed by biparametric FACS analysis. Three x10^5^ A-498 and Caki-2 RCC cells were treated with axitinib or with vehicle for up to 96 h. The percentage of positive cells determined over 10,000 events was analyzed on a FACScan cytofluorimeter using the CellQuest software.

### DNA fragmentation assay

RCC cells were treated as above described for up to 96 h, and genomic DNA was extracted using a DNA extraction kit (Qiagen). The purified samples were then subjected to electrophoresis on a 1.25% agarose gel, and DNA was stained with ethidium bromide. Ultraviolet spectroscopy at 302 nm was used to obtain the results.

### Reactive Oxygen Species (ROS) production

Cells were cultured for up to 96 h with axitinib or vehicle. Cells were washed with PBS, pulsed with DCFDA for 10 min at 37°C, 5% CO_2_, and analyzed by FACScan cytofluorimeter using the CellQuest software.

### α-Tubulin and nuclei staining

Cells cultured as above described were fixed in 2% formaldehyde and 0.5% triton X-100 for 10 min at room temperature and then in 4% formaldehyde and 0.5% triton X-100 for 10 min. To examine the expression of α-tubulin, fixed cells were permeabilized in 0.1% Tween-20/3% BSA, and stained with mouse anti-α-tubulin antibody (1:50). Cells were further incubated with Alexa-594-conjugated GAM (1:100). For nuclei analysis, cells were fixed in Carnoy's fixative (1:3 glacial acetic acid: absolute methanol) and stained with 0,05 μg/ml Hoechst 33258. Stained cells were examined by using the Olympus BX51 microscope.

### Immunofluorescence and microscopic analysis

For immunofluorescence analysis, RCC cells were fixed with 1:1 MeOH, acetone for 20 min at −20°C and labeled with anti-8-hydroxyguanine (8-oxo-dG) antibody (1:250) diluted in 1X PBS containing 1% BSA, 0.01% Tween 20 at 4°C o/n in a humidified chamber according to manufacturer's instructions. Cells were further incubated with Alexa-594-conjugated GAM (1:100). Photographs were acquired and analyzed by an Olympus BX51 microscope.

### Cytofluorimetric analysis

NKG2D and DNAM-1 ligand surface expression on Caki-2 and A-498 cells was analyzed by immunofluorescence staining using anti-MICA, anti-MICB, anti-ULBP1/2,5,6/3, anti-PVR or anti-nectin-2 APC-conjugated mAbs or relative APC-conjugated IgG isotypes according to manufacturer's instructions. In some experiments, RCC cells were double stained with anti-MICB APC-conjugated mAb and C_12_FDG or with anti-MICB, anti-MICA, anti-ULBP1 APC-conjugated mAbs and PI. Fluorescence was analyzed by FACScan cytofluorimeter using the CellQuest software.

### Degranulation assay

NK cell-mediated cytotoxicity was evaluated using the degranulation lysosomal marker CD107a as described [42]. As source of effector cells freshly purified NK cells were used. Peripheral blood mononuclear cells (PBMC) were separated from buffy coats of healthy donors by Lymphoprep (Nycomed, Oslo, Norway) gradient centrifugation. Freshly isolated NK cells were then isolated from PBMC by negative selection using a magnetically activated cell sorter NK isolation kit (Miltenyi Biotec, Bologna, Italy). This purification protocol resulted in a purity of more than 95% of negatively selected NK cells. After 72 h treatment with axitinib, RCC cells were incubated with NK cells at effector:target (E:T) ratios of 1:1 in a flat-bottom 96-well tissue culture plate in complete medium. In some experiments axitinib-treated where exposed to NAC (10 mM, for 1 h). The plates were then incubated at 37°C in a 5% CO_2_ atmosphere for 2 h. Thereafter, cells were incubated with anti-CD107a/APC (or cIgG/APC) for 45 min at 4°C. Cells were also stained with anti-CD56/PE to gate NK cell population.

### Statistical analysis

The data presented represent the mean and standard deviation (SD) of at least 3 independent experiments. The statistical significance was determined by Student's t-test and by one way ANOVA; *,# *p* < 0.01. The statistical analysis of IC_50_ levels was performed using Prism 5.0a (Graph Pad).

## SUPPLEMENTARY MATERIAL FIGURES


