# Supplementary material for: Axitinib induces DNA damage response leading to senescence, mitotic catastrophe, and increased NK cell recognition in human renal carcinoma cells
Source: Oncotarget. 2015 Oct 14;6(34):36245–59. doi: 10.18632/oncotarget.5768 (PMC4742174; doi:10.18632/oncotarget.5768)
Supplement: Supplementary file 1 [file oncotarget-06-36245-s001.pdf]

Axitinib induces DNA damage response leading to senescence, mitotic catastrophe, and increased NK cell recognition in human renal carcinoma cells

Supplementary Material

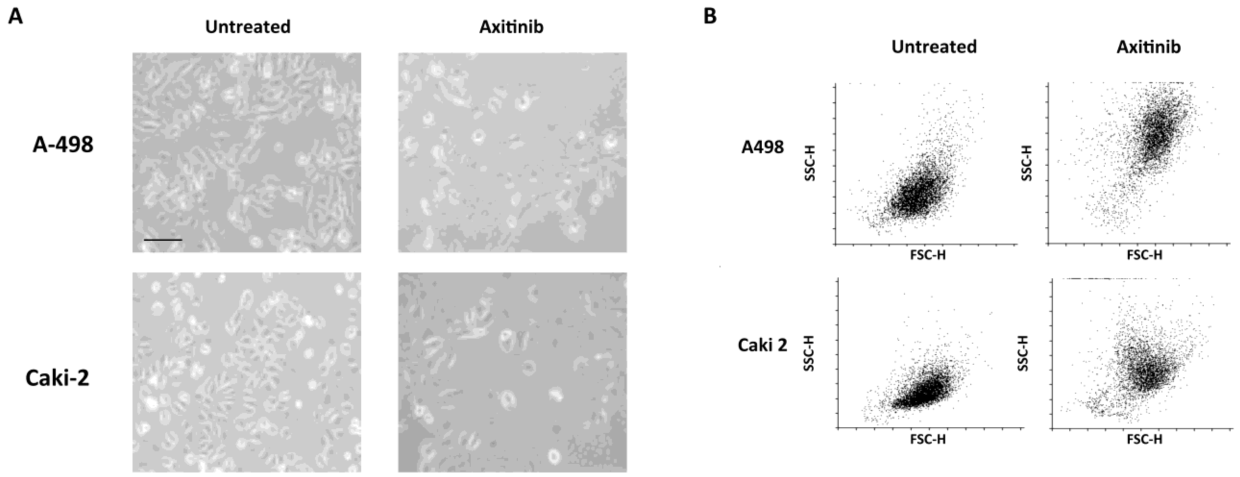

Supplementary Fig. 1 Morphology of axitinib treated RCC cell lines

A) Morphological examination of axitinib treated RCC cells after 72 h. Bar: 100 μM.

B) Representative forward-scatter (FSC) and side-scatter (SSC) dot plots after 72 h of treatment.

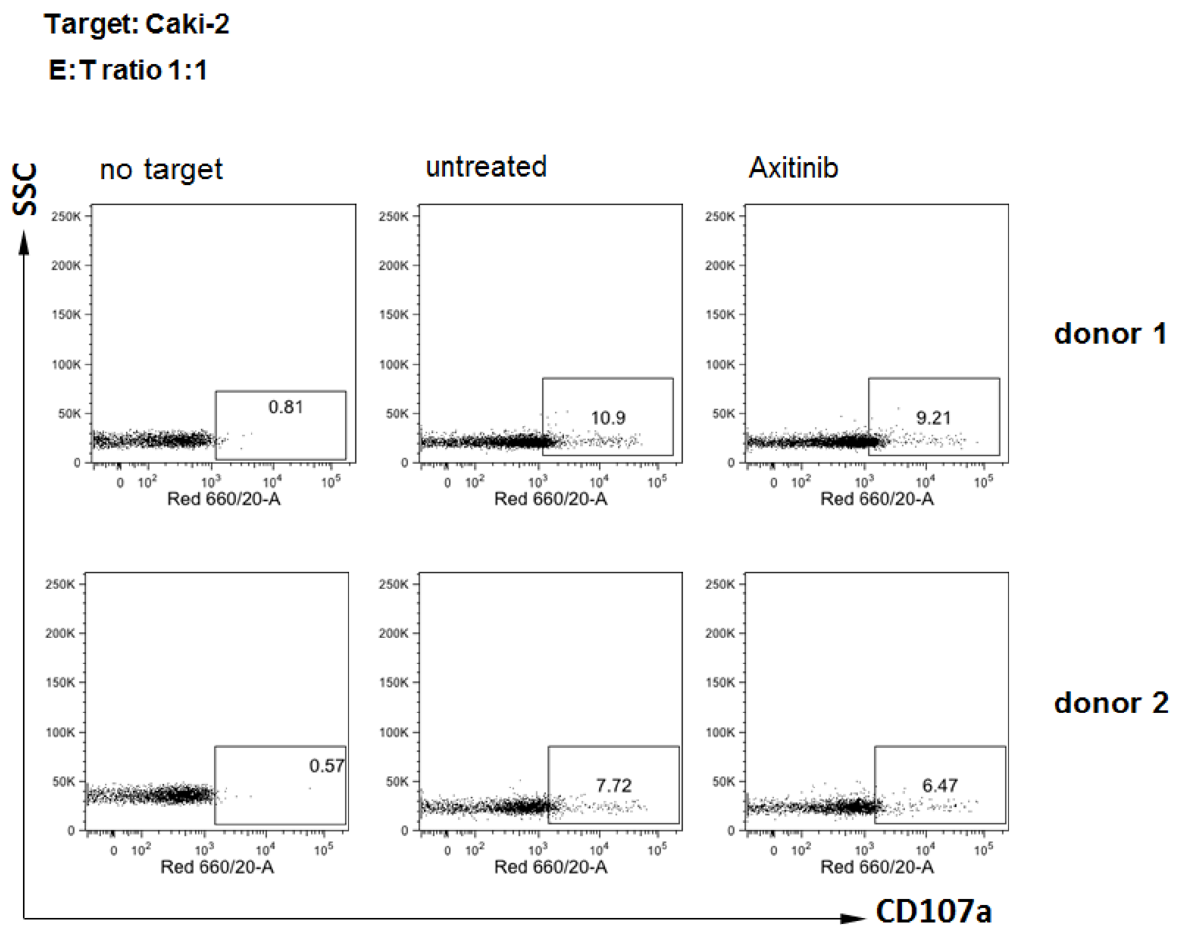

Supplementary Fig. 2 Axitinib does not stimulate NK cell degranulation in Caki-2 RCC cells.

Caki-2 RCC cells were treated with axitinib 25 μM for 72 h and then incubated with freshly isolated human peripheral blood NK cells from two different healthy donors at 1:1 ratio for 2 h. Results are expressed as percentage of $CD107a^+$ NK cells.
